# A comparative study on the efficacy of a retrograde perfusion technique and an antegrade perfusion technique for donor kidney recovery in transplantation in pigs

**DOI:** 10.1186/s12893-017-0285-z

**Published:** 2017-08-03

**Authors:** Xiuwu Han, Xuhui Zhu, Tao Li, Yansheng Li, Hui Shan, Peng Zhang, Bulang He

**Affiliations:** 1 Department of Urology, Beijing Chaoyang Hospital, Affiliated to Capital Medical University, Beijing, 100043 People’s Republic of China; 2 Liver and Kidney Transplant Service,Sir Charles Gairdner Hospital, School of Surgery, The University of Western Australia, Nedlands, Perth, WA 6009 Australia

**Keywords:** Kidney transplantation, Organ recovery, Retrograde perfusion, Antegrade perfusion, Experiment, Pig

## Abstract

**Background:**

Donor organ shortage is a significant problem in kidney transplantation. Improvement of perfusion techniques can increase the number of available organs. The aim of this study is to investigate the efficiency and safety of retrograde perfusion (RP) of kidney grafts during organ recovery after transplantation in pigs.

**Methods:**

Ten pigs were divided into two groups, six in the study group for the RP technique and four in the control group for standard antegrade perfusion (AP). The left kidney was removed and perfused by the RP or AP method according to the study group. The perfused left kidney was auto-transplanted to the right groin location. The right kidney was removed and perfused in the same manner and then stored at 4 °C for 24 h prior to histopathological analysis. Data in both groups were observed and recorded.

**Results:**

All kidneys perfused by both the RP and AP methods were satisfactory in appearance. All grafts showed diuresis from the first postoperative day onward. On postoperative day 7, the mean serum creatinine (Scr) and blood urea nitrogen (BUN) levels were 174 ± 9.7 ìmol/L and 27.7 ± 2.5 mg/dL in the RP group, and they were 168 ± 13.7 ìmol/L and 26.5 ± 4.3 mg/dL, respectively, in the AP group (*p* = 0.483 for Scr and *p* = 0.646 for BUN). The mean peak Scr levels in the RP group (570 ìmol/L) and the AP group (530 ìmol/L) were similar. All pigs survived with adequate renal function throughout the study period. There was minimal interstitial and tubular edema, and there was endothelial cell swelling in some specimens before revascularization in both groups. At postoperative day 7, the auto-transplanted kidneys showed normal glomerular and tubular structure with little interstitial edema and inflammatory cell infiltration in the grafts. No differences were identified between the two groups. Under electron microscopy, the tubular epithelial cells, glomeruli, and glomerular capillary endothelium of the grafts appeared normal in both groups after 24 h in cold storage.

**Conclusions:**

Kidney grafts in pigs perfused by RP had normal function after transplantation compared with the AP control group. Therefore,retrograde perfusion is potentially an efficient, safe kidney perfusion method for organ recovery.

**Electronic supplementary material:**

The online version of this article (doi:10.1186/s12893-017-0285-z) contains supplementary material, which is available to authorized users.

## Background

Because donor organ shortage is a significant problem in kidney transplantation, all efforts should be made to use any available donor organs. Potential kidney grafts, especially from “donation after cardiac death” (DCD) donors, may be discarded if they are not adequately perfused. Improvement of perfusion techniques can reduce the number of discarded donor kidneys and increase the number of available organs. Retrograde perfusion (RP) through the inferior vena cava has been performed in some cardiothoracic operations to protect the abdominal viscera and kidneys [1–4]. In a previous study, we demonstrated the feasibility and efficiency of kidney graft RP using rabbit and sheep models [5]. We performed 24 kidney transplantations with kidneys lavaged using the RP technique, which treated renal artery variations or injury, and we obtained ideal clinical results [6]. In that study the RP method was mostly used after conventional antegrade perfusion (AP) had failed during kidney harvesting. All donor kidneys in the RP group had vascular malformation or damage. These circumstances might have caused bias in the clinical observations. A comparative functional recovery model study on the RP technique has not been attempted. The aim of this study is to investigate the efficiency of kidney graft RP after transplantation in pigs. Using this preclinical large animal model, we sought evidence for clinical application of kidney graft RP, particularly when standard renal artery perfusion is difficult or impossible.

## Materials and methods

### Animal preparation

All animal experiments were approved by the animal ethics committee of Beijing Chaoyang Hospital. Ten white female pigs, weighing 40–45 kg, were transported to the large animal experimental laboratory and acclimated for 2 days prior to the experiment. Pigs were housed in pens under standard conditions. A standard diet was maintained with unlimited access to tap water. The urine volume and serum creatinine levels were recorded for each pig before the study.

### Anesthesia for surgery

Pigs were fasted for 12 hours prior to surgery. Each pig was weighed and premedicated with a tiletamine-zolazepam mixture (Virbac, Carros, France) (4.4 mg/kg) and xylazine (Sigma, St Louis, MO, USA) (2.2 mg/kg), which were administered intramuscularly. Ear vein cannulation was established for intravenous administration of propofol (1 mg/kg) to facilitate intubation. Oral intubation was performed with a cuffed endotracheal tube. Following successful intubation, adequate surgical anesthesia was maintained using inhaled isoflurane. The criteria for adequate anesthesia included a lack of muscle tone, absence of spontaneous ventilatory effort, and dilation of pupils. The ventilator settings were on the volume control mode with a tidal volume of 10–15 mL/kg and peak inspiratory pressure below 25 cm H_2_O. The ventilation rate was adjusted to keep the end-tidal CO_2_ between 35 and 45 mm Hg. Body temperature was measured by insertion of a rectal probe and was maintained between 36°C and 38°C using heating pads. Hartmann’s fluids were given via central a vein catheter (10mL/kg/h) throughout surgery. The electrocardiogram and oxygen saturation were monitored throughout the procedure.

### Nephrectomy

Surgical procedures were performed under sterile conditions. Prior to surgery, heparin (3,000 IU, intravenously) was given to prevent blood clots, and ampicillin (1,000 mg, intravenously) was prophylactically given to prevent infection. A low ventral midline incision was made for nephrectomy. The left kidney was removed after dissecting the retroperitoneum, maintaining blood supply to the ureter. The renal vascular pedicle of the graft was kept as long as possible by dissecting the renal artery and vein up to the aorta and vena cava. First, the ureter was divided; then, the renal artery and vein were cut and the kidney was removed. The warm ischemia time was defined as the time of application of a clamp to the vascular artery until initiating perfusion of the kidney. The cold ischemia time was defined as the time from initiating perfusion of the kidney to reperfusion of the kidney after revascularization. The right kidney was kept in storage for 24 hours at 4°C for histopathological analysis.

Ten pigs were divided into two groups: six in the study group for RP and four in the control group for standard AP. The left kidney was removed and perfused by the RP or AP methods according to the study group. The perfused left kidney was auto-transplanted to the right groin location. After auto-transplantation, the right kidney was removed and perfused in the same manner; then, it was stored at 4°C for 24 hours prior to histopathological analysis.

### The study group: RP of kidney graft

Immediately after removal, kidneys were perfused retrogradely through the vein with hypertonic citrate adenine (HCA;Changzheng Hospital, Shanghai, China) fluid at 4°C under 60 cm H_2_O pressure. Perfusion was interrupted after 40 mL was injected to allow perfusate to flow back out of the kidney through the renal vein. The RP technique is shown in the Additional file 1: Video S1. The kidney was completely lavaged to a pale color after this maneuver was repeated four to five times. Outflow at the arterial end and back flow at the renal vein were clear. The total perfused volume was approximately 200 mL.


Additional file 1: Video S1. Example video file of RP technique for donor kidney lavage. (MP4 56767 kb)


### The control group: AP of kidney graft

Immediately after removal, kidneys were perfused antegradely through the renal artery with HCA fluid at 4°C under 100 cm H_2_O pressure. The perfusion was continuous until the outflow at the venous end was clear, which is consistent with our procedure in clinical practice. The volume required for this perfusion was also approximately 200 mL.

### Surgery for auto-transplantation

After a short period of cold preservation(2-4 hours), the perfused left kidney was auto-transplanted to the right groin location using the same midline incision. The procedures for auto-transplantation were the same in both groups. The renal vein was anastomosed end-to-side with the vena cava. The renal artery was anastomosed end-to-side with the common iliac artery. Prolene 6-0 sutures were used for both vessels. Reperfusion was established by first releasing the venous clamp and then the arterial clamp. One hundred milliliters of 20% mannitol was given intravenously before finishing the anastomosis. During these procedures, the kidney was cooled in a specially designed disposable gauze bag to eliminate the second warm ischemia. The home-made bag, which has been used in our center for almost three decades, consists of three compartments. The middle compartment holding the graft is surrounded by two external compartments filled with ice flakes (sterile saline ice). After placing the graft in the middle compartment, the renal vein and artery were pulled outside the bag via a small hole in the middle compartment while ensuring the bag was filled with ice. The ureter was cannulated by a silicon tube or a 10-Fr catheter, and then tunneled through the abdominal wall for continuous observation of urine output from the renal graft. The abdomen was closed. The pig was returned to its pen after recovery from anesthesia.

Needle biopsies of kidney grafts were taken with 16-gauge Tru-Cut needles at different time points: immediately after perfusion, before revascularization, after 24 hours storage, and on day 7 following transplantation. Tissue samples were fixed in 4% buffered formalin for light microscopy. For electron microscopy, samples were postfixed in 1% osmium tetroxide and embedded in Epon 812. Ultrathin sections were stained with uranyl acetate and lead citrate, and they were examined under an electron microscope (H-600; Hitachi, Tokyo, Japan).

After surgery, pigs were allowed to have free access to food and water. Analgesia was administered for up to 72 hours according to approved protocols. Heparin (1500 IU/day) was given subcutaneously. At day 7 after transplantation, the pigs were anesthetized and laparotomy was performed to remove the transplanted kidney. The pigs were then euthanized by intravenous injection of 20 mL of 10% potassium chloride.

### Peri- and postoperative monitoring

Pigs were monitored closely according to the study protocol. Urine output was recorded daily. Blood samples were taken from the venous catheter daily to measure the serum creatinine (Scr). The venous catheters were flushed daily with heparinized saline (10 IU/mL) and sealed to prevent blood clot formation in the catheters. The kidneys were examined after both perfusion and graft nephrectomy. Light and electron microscopy were used for histopathological analysis of kidney graft biopsies. The method used for assessment of histological parameters was similar to that described by Hosgood et al. [7]. The sections of kidney specimens were scored 20 fields at 400× magnification, assessing changes in the following morphologic variables: brush border loss, tubular dilation, tubular debris, vacuolation, interstitial edema, basement membrane, tubular epithelial loss, glomerular shrinkage, erythrocyte count, apoptotic/pyknotic nuclei, and lymphocytic infiltrate. Samples were scored from 0 to 3 according to the level of damage, with 0 representing normal, 1 mild, 2 moderate, and 3 severe morphologic changes.

**Fig. 1:**
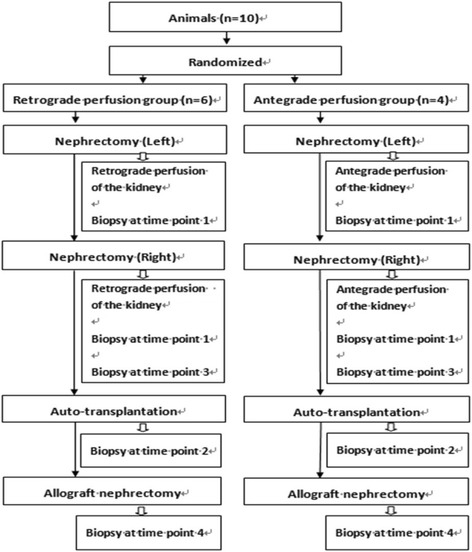
The diagram of the experimental procedures and time points of biopsy. Time points for biopsy: 1. Immediately after perfusion, 2. Before revascularization, 3. After 24 h of storage, and 4. On day 7 following transplantation before graft removal

The histopathological examination was performed on a blinded basis by an experienced histopathologist. The diagram of the experimental procedures and time points of biopsy are shown in Fig 1.

### Statistical analysis

Data are expressed as the mean ± standard deviation. The student’s *t*-test and Mann–Whitney *U*-test were used to assess the statistical significance of differences between the two groups. All statistical calculations were performed using SPSS software (Statistical Package for the Social Sciences Version 17.0 for Windows, 2001; SPSS, Chicago, IL, USA). *P* < 0.05 was considered statistically significant.

## Results

**Fig. 2:**
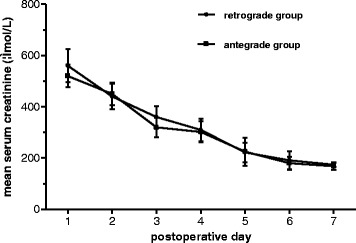
Postoperative serum creatinine levels in the two groups

There were no significant differences in the warm ischemia time, cold ischemia time, perfusion time, or amount of perfusion fluid between the two groups (Table 1). Upon gross examination, all kidneys perfused by both RP and AP were satisfactory in appearance. All grafts showed diuresis from the first postoperative day onward, which indicated that all had immediate function. Urine output was observed soon after reperfusion during the operation. There was no significant difference in the daily urine output between the two groups. The preoperative baseline Scr and BUN values were 132 ± 12 ìmol/L and 14 ± 2.5 mg/dL,respectively in the RP group, and 128 ± 23 ìmol/L and 14 ± 2.8 mg/dL, respectively in the AP group. On postoperative day 7, the mean Scr and BUN levels were 174 ± 9.7 ìmol/L and 27.7 ± 2.5 mg/dL, respectively in the RP group, and 168 ± 13.7 ìmol/L and 26.5 ± 4.3 mg/dL respectively, in the AP group. Differences between the RP and AP groups were not statistically significant (*p* = 0.483 for Scr and *p* = 0.646 for BUN).The mean peak serum creatinine levels of RP (570 ìmol/L) and AP (530 ìmol/L) were similar. All pigs survived with adequate renal function throughout the study period (Table 1 and Fig 2). No surgical complications were encountered during the study.

**Table 1:** The observation data for the two groups

Variables	Retrograde group	Antegrade group	*p* value
Number (n)	6	4	
Perfusion time (min)	7.1 ± 1.6	7.6 ± 1.1	0.585
Amount of perfusion fluid (ml)	194 ± 20	203 ± 13	0.442
First warm ischemia time (min)	3.0 ± 0.3	3.3 ± 0.5	0.527
Cold ischemia time (min)	57 ± 12	55 ± 13	0.669
Operation time(h)	3.3 ± 0.5	3.0 ± 0.5	0.326
Urine output at 7 day(ml)	1206 ± 316	1307 ± 398	0.672
Scr at 7 day (μmol/dl)	174 ± 9.7	168 ± 13.7	0.483
BUN at 7 day (mg/dl)	27.7 ± 2.5	26.5 ± 4.3	0.646

*Scr* serum creatine and *BUN* blood urea nitrogen

### Histopathology under light microscopy

**Fig. 3:**
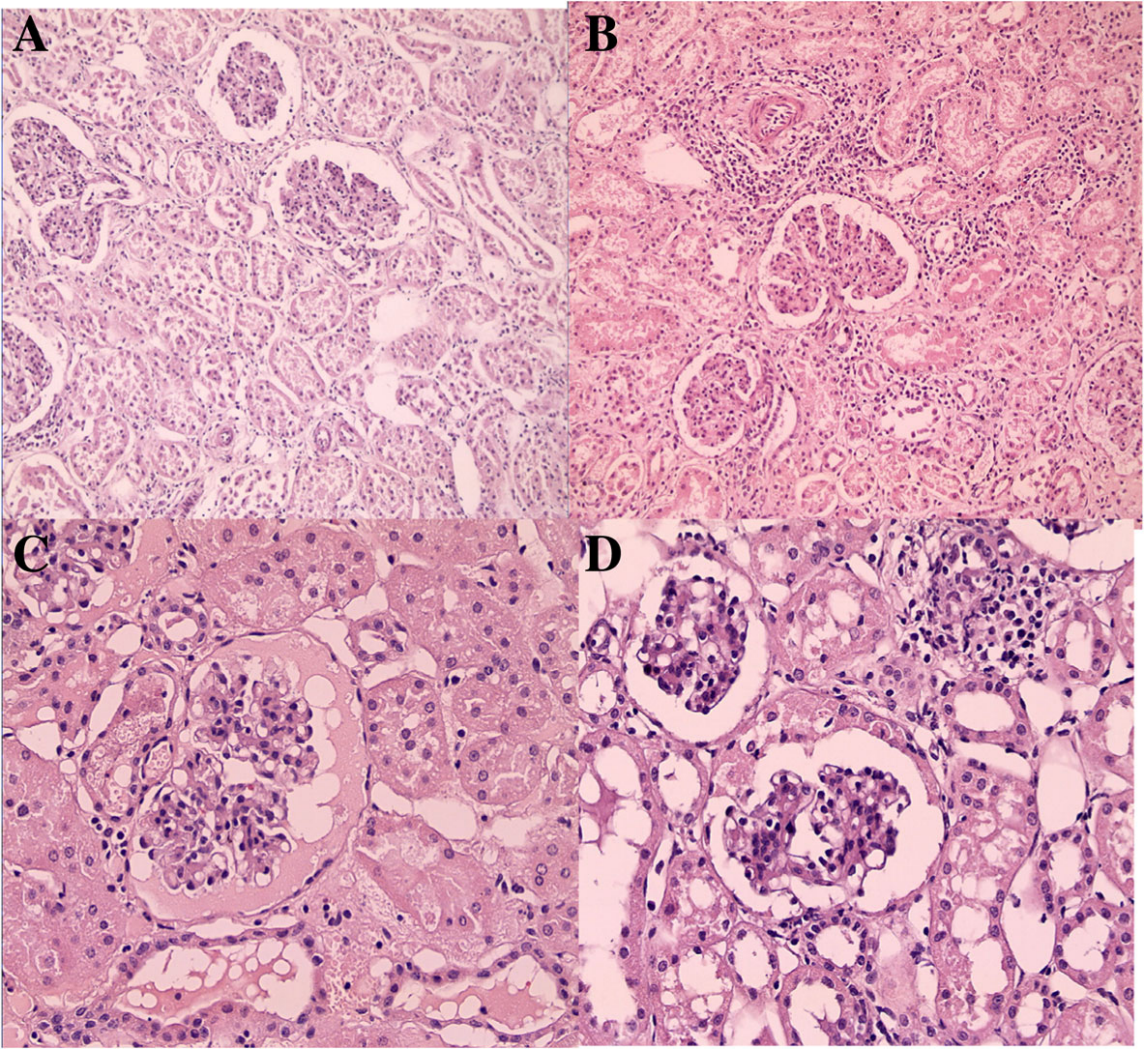
Both retrograde perfused kidneys (**a**) and antegrade perfused kidneys (**b**) have minimal interstitial, tubular edema and endothelial cell swelling with good preservation of renal architecture at 2–4 h in cold storage (hematoxylin and eosin staining, 20×). Retrograde perfusion (**c**) resulted in glomerular shrinkage with effusion and antegrade perfusion (**d**) resulted in glomerular shrinkage at the end of 24 h in cold storage (hematoxylin and eosin staining, 40×)

**Fig. 4:**
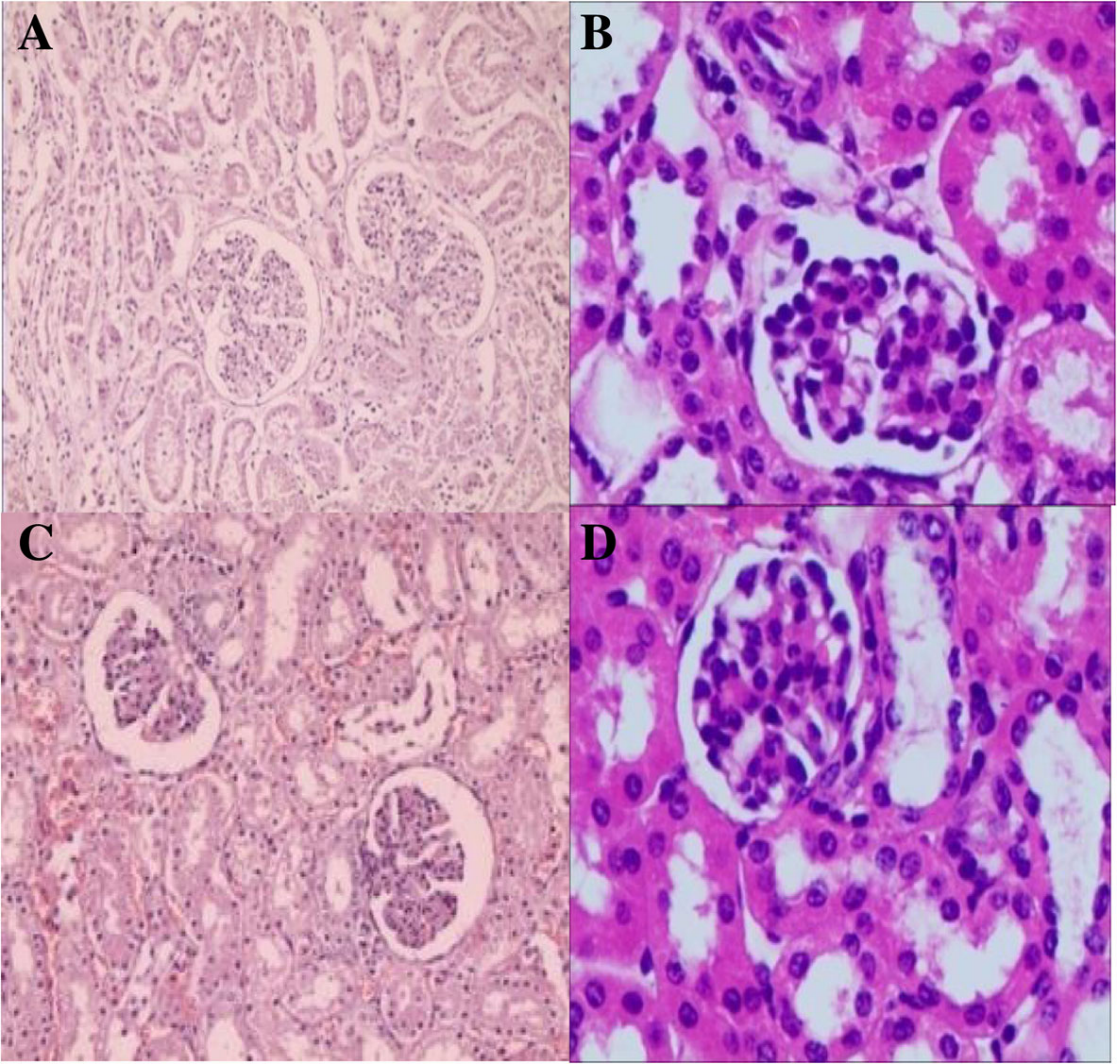
At postoperative day 7, the grafts showed near normal glomerular and tubular structure, with little interstitial edema and inflammatory cell infiltration, but no differences were identified between the RP (**a** and **b**, hematoxylin and eosin staining, 20×, 40×) and AP groups (**c** and **d**, hematoxylin and eosin staining, 20×, 40×)

No signs of vascular, glomerular, or tubular damage were observed by light microscopy immediately after perfusion in either group. As shown in Fig 3, there was minimal interstitial and tubular edema, and there was endothelial cell swelling in some specimens before revascularization in both groups, but these could not be differentiated between the groups. The severity and occurrence of tubular degeneration was slightly and equally augmented in both groups after 24 hours in cold storage (Table 2). At postoperative day 7, the auto-transplanted kidneys had a near-normal glomerular and tubular structure, with little interstitial edema and inflammatory cell infiltration in the grafts, but no differences were identified between the RP and AP groups(Table 3 and Fig 4).

**Table 2:** Histology data from 24 h postpreservation biopsies for the two groups

Parameters	Retrograde group	Antegrade group	*P* value
Tubular dilatation	1.15 ± 0 51	1.60 ± 057	0240
Brush border loss	0 81 ± 0 34	1.25 ± 034	0109
Epithelial loss	1.23 ± 042	1.40 ± 036	0454
Tubular debris	088 ± 056	1.05 ± 058	0669
Vacuolation	098 ± 034	092 ± 034	0667
Base membrane loss	063 ± 025	060 ± 014	0747
Interstitial edema	178 ± 040	190 ± 082	1.000
Glomerular shrinkage	197 ± 061	1 90 ± 050	0748
Glomerular perfusion^a^	1.53 ± 0·81	2.07 ± 0·298	0.134
glomerular effusion	1.68 ± 0·55	0.80 ± 0·64	0.054
Apoptotic/pyknotic nuclei	0.15 ± 0·25	0.25 ± 0·38	0.631

Values are the mean ± SD.The Mann-Whitney U- test was used to assess the statistical significance of differences between the two groups.^a^ Erythrocyte count

Samples were scored from 0 to 3 according to the level of damage with 0 representing normal, 1 mild, 2 moderate, and 3 severe morphologic changes

**Table 3:** Histology data from 7 day autotransplant allograft kidney biopsies for the two groups

Parameters	Retrograde group	Antegrade group	*P* value
Tubular dilatation	1.10 ± 011	1.12 ± 050	0220
Brush border loss	112 ± 031	1.21 ± 032	0100
Epithelial loss	1.25 ± 0·40	1.30 ± 031	0463
Tubular debris	079 ± 033	0.75 ± 051	0·607
Vacuolation	085 ± 023	076 ± 032	0707
Base membrane loss	056 ± 020	059 ± 032	0806
Interstitial edema	269 ± 066	280 ± 062	1.090
Glomerular shrinkage	208 ± 081	230 ± 052	0701
lymphocytic infiltrate	3.98 ± 0·50	3.81 ± 0·43	0.067
Apoptotic/pyknotic nuclei	0.18 ± 0·34	0.20 ± 0·45	0.761

Values are the mean ± SD. The Mann-Whitney U- test was used to assess the statistical significance of differences between the two groups. Samples were scored from 0 to 3 according to the level of damage with 0 representing normal, 1 mild, 2 moderate, and 3 severe morphologic changes

### Histopathology under electron microscopy

**Fig. 5:**
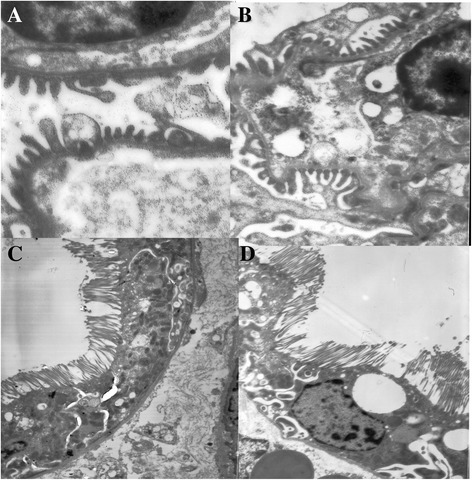
Electron microscopy showed glomerular capillary endothelium and tubular epithelial cells were of normal appearance in the retrograde group (**a**: ~20,000, **c**: ~4000) and the antegrade group (**b**: ~12,000, **d**: ~6000) at the end of 24 h in cold storage

Transmission electron microscopy was used to examine the ultrastructure of the perfused kidneys after 24 hours in cold storage. In both groups the tubular epithelial cells, glomeruli, and glomerular capillary endothelium of the graft appeared near normal (Figure 5).

## Discussion

Adequate organ perfusion and preservation are critical steps in organ transplantation. In preparation for transplantation, kidneys usually undergo AP from the artery to vein. However, AP cannot be used effectively when donor kidneys are subject to special circumstances, such as artery spasm or intraoperative damage to the renal artery. In such cases, RP through the renal vein is the easiest way to adequately perfuse the donor kidney [5, 6]. It has been demonstrated that kidney perfusion can be conducted by retrograde flow from the efferent to the afferent arterioles [8]. RP via the inferior vena cava has been conducted in some cardiothoracic operations to protect the abdominal viscera, including the kidneys [1–4]. These studies have shown the feasibility and safety of RP of the kidneys.

Our previous study in rabbits showed that all kidney grafts were well preserved for 24 hours after RP [5]. Zhao et al. [9] reported that RP of rabbit kidneys in vivo caused less injury and led to faster recovery from warm ischemic injury than AP. Unlike previous studies, the perfused kidneys were auto-transplanted for functional recovery model investigation after RP or AP. We performed RP in an interrupted fashion to lavage the renal graft rather than a continuous technique, which was combined with lower perfusion pressure, to lessen hydrostatic mechanical damage. To evaluate the effects of the RP technique itself on renal graft function after auto-transplantation, we restricted the warm ischemic time and graft preservation time to as short as possible before auto-transplantation. Kidney grafts lavaged by RP had normal function after transplantation compared with the AP control group. No adverse events were identified. Light and electron microscopy showed that the RP and AP histopathologies were similar. Renal veins have less variation and a greater caliber than renal arteries, and the renal vein system has extensive communicating branches and no valves in the renal parenchyma [1, 2, 10]. Therefore, from an anatomical point of view RP may be a reasonable way to perfuse kidneys. Clinical application of kidney graft RP could be considered, particularly under circumstances when standard renal artery perfusion is difficult or impossible. Currently, kidney grafts from DCD donors are increasingly used. The problems for these kidney grafts are that the warm ischemia time is prolonged and anticoagulation agents should not be administered. The results of an initial flush of these kidney grafts are crucially important. According to our study results, we surmise that RP may play a role in increasing the utility rate of kidney grafts from DCD donors.

## Conclusion

RP is an efficient kidney perfusion method for organ recovery in the initial flush of donor kidneys in a porcine model, and it holds promise as a clinical perfusion technique in kidney transplantation.

## Abbreviations

AP: Antegrade perfusion
BUN: Blood urea nitrogen
DCD: Donation after cardiac death
HCA: Hypertonic citrate adenine
RP: Retrograde perfusion
Scr: Serum creatinine
